# Genetic and phenotypic analysis of 225 Chinese children with developmental delay and/or intellectual disability using whole-exome sequencing

**DOI:** 10.1186/s12864-024-10279-1

**Published:** 2024-04-22

**Authors:** Heqian Ma, Lina Zhu, Xiao Yang, Meng Ao, Shunxiang Zhang, Meizhen Guo, Xuelin Dai, Xiuwei Ma, Xiaoying Zhang

**Affiliations:** 1 The School of Public Health, Guilin Medical University, 1 Zhiyuan Road, Lingui District, 541199 Guilin, PR China; 2 Faculty of Pediatrics, The Chinese PLA General Hospital, 100700 Beijing, China; 3 National Engineering Laboratory for Birth Defects Prevention and Control of Key Technology, 100700 Beijing, China; 4 Beijing Key Laboratory of Pediatric Organ Failure, 100700 Beijing, China; 5 The Guangxi Key Laboratory of Environmental Exposomics and Entire Lifecycle Heath, 1 Zhiyuan Road, Lingui District, 541199 Guilin, PR China; 6 Guangxi Health Commission Key Laboratory of Entire Lifecycle Health and Care, 1 Zhiyuan Road, Lingui District, 541199 Guilin, PR China

**Keywords:** Developmental Delay, Intellectual disability, Children, Whole-exome sequencing, Brainstem auditory evoked potential, Hearing loss, Phenotype

## Abstract

Developmental delay (DD), or intellectual disability (ID) is a very large group of early onset disorders that affects 1–2% of children worldwide, which have diverse genetic causes that should be identified. Genetic studies can elucidate the pathogenesis underlying DD/ID. In this study, whole-exome sequencing (WES) was performed on 225 Chinese DD/ID children (208 cases were sequenced as proband-parent trio) who were classified into seven phenotype subgroups. The phenotype and genomic data of patients with DD/ID were further retrospectively analyzed. There were 96/225 (42.67%; 95% confidence interval [CI] 36.15–49.18%) patients were found to have causative single nucleotide variants (SNVs) and small insertions/deletions (Indels) associated with DD/ID based on WES data. The diagnostic yields among the seven subgroups ranged from 31.25 to 71.43%. Three specific clinical features, hearing loss, visual loss, and facial dysmorphism, can significantly increase the diagnostic yield of WES in patients with DD/ID (*P* = 0.005, *P* = 0.005, and *P* = 0.039, respectively). Of note, hearing loss (odds ratio [OR] = 1.86%; 95% CI = 1.00-3.46, *P* = 0.046) or abnormal brainstem auditory evoked potential (BAEP) (OR = 1.91, 95% CI = 1.02–3.50, *P* = 0.042) was independently associated with causative genetic variants in DD/ID children. Our findings enrich the variation spectrums of SNVs/Indels associated with DD/ID, highlight the value genetic testing for DD/ID children, stress the importance of BAEP screen in DD/ID children, and help to facilitate early diagnose, clinical management and reproductive decisions, improve therapeutic response to medical treatment.

**Supplementary Information:**

The online version contains supplementary material available at 10.1186/s12864-024-10279-1.

## Introduction

Developmental delay or intellectual disability (DD/ID) is one of the most common neurodevelopmental disabilities with high clinical heterogeneity [1, 2]. It is often classified as either isolated or syndromic DD/ID, and syndromic DD/ID patients present with additional clinical manifestations, such as congenital anomalies, dysmorphic features, epilepsy or unusual behavior [3, 4]. DD/ID affects 1–2% of children worldwide and pose heavy medical, psychological, financial, and social burden [5, 6].

DD/ID might be caused by environmental factors, such as gestational substance abuse, birth complications, infections, and traumas [7, 8]. DD/ID can also be caused by genetic factors, more than 700 genes have been identified to date [9–13]. As genomic technologies progress, new DD/ID genes can be identified rapidly. Whole-exome sequencing (WES) mainly focuses the detection of single nucleotide variants (SNVs) and small insertions/deletions (Indels), which has been proven to result in a high overall diagnostic yield of 30–40% in patients with DD/ID [12, 14–17]. In 2021, the American College of Medical Genetics and Genomics (ACMG) strongly recommended WES as a first- or second-tier tool for diagnosis of DD/ID to reduce “diagnostic odyssey” [12]. Furthermore, the results of WES may lead to earlier diagnosis, improve therapeutic response, facilitate clinical management, and impact reproductive decisions [12, 15]. Therefore, the objective of this study was to determine the diagnostic yield of DD/ID by WES, to better characterize the genetic landscape of DD/ID and to determine whether WES results can impact medical management.

Furthermore, there is a paucity of information about associations of clinical manifestations with identified causative variants for DD/ID. According to previous studies, the diagnostic rates of WES for isolated and syndromic DD/ID were equivalent [1, 4, 16, 17],, while a meta-analysis reported that the diagnostic yield was 54% for syndromic DD but 31% for isolated DD [18]. Furthermore, several studies have shown that specific clinical features, such as craniofacial anomalies and abnormal head circumference, can increase the diagnostic yield of WES in patients with DD/ID, but none of these impacts are statistically significant [4, 16]. However, Michelle VS et al. reported that the diagnostic yield of WES was significantly greater in DD patients with dysmorphic features than in patients without dysmorphic features [14]. Therefore, another objective of this study was to determine whether specific clinical features can increase the genetic diagnostic yield of DD/ID, and to highlight the importance of routine physiological and biochemical tests in genomic screens.

## Methods

### Study participant

**Fig. 1:**
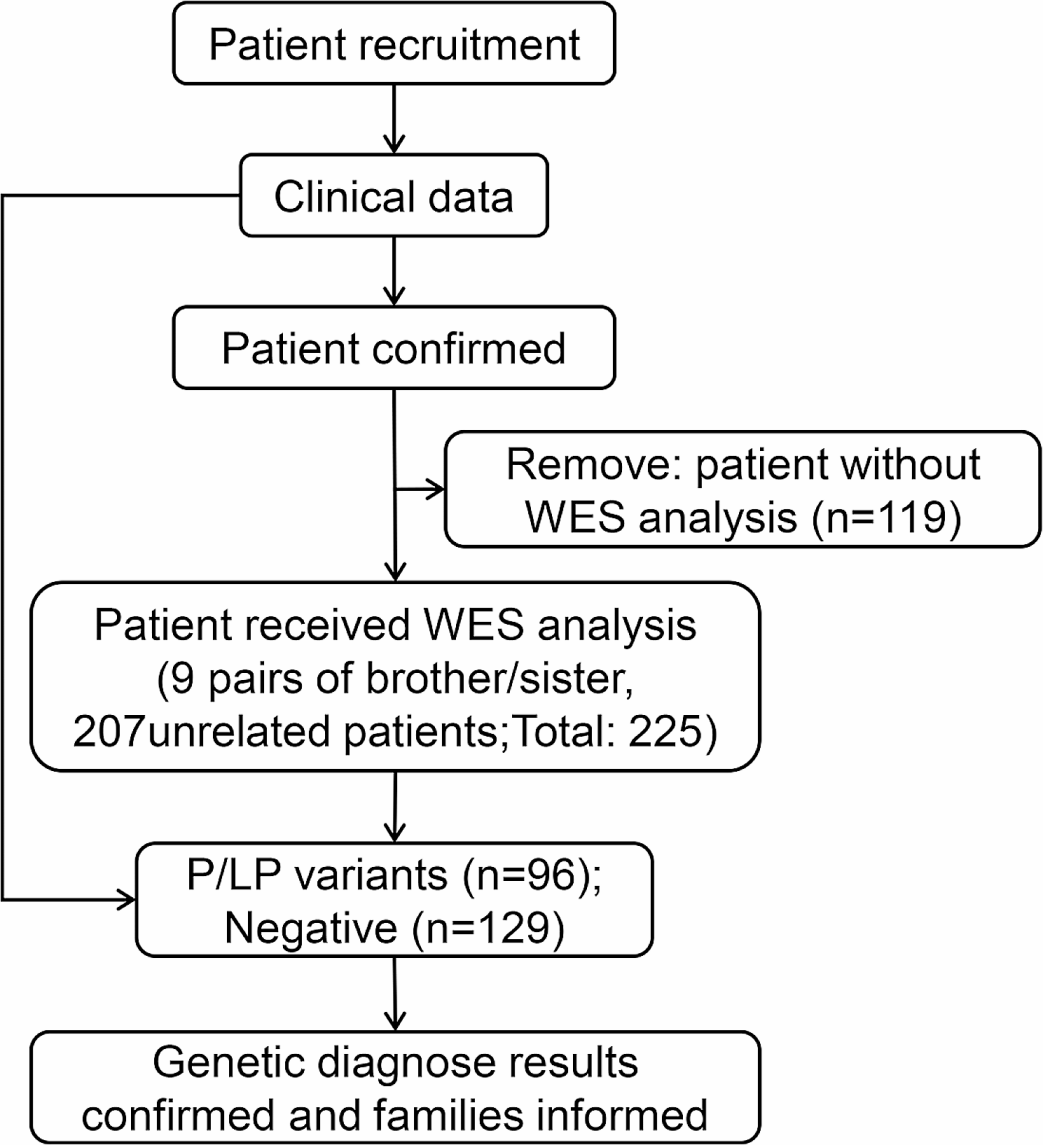
Flow chart of the study desig

As illustrated in Fig. 1, this study recruited 225 DD/ID children after obtaining signed informed consents from their parents or legal guardians, between March 2018 and December 2021 in Seventh Medical Center of PLA General Hospital. The detailed clinical data (e.g. age, gender, perinatal history, birth history, neurodevelopmental history, family history) and clinical examinations data (such as physiological testing, biochemical testing) of all patients were reviewed. The exclusion criteria were as follows: (1) parents/guardians refused to sign informed consents; (2) children had nervous system infections or traumas; (3) maternal substance abuse or infections; (4) birth complications; (5) incomplete medical records; (6) positive karyotype test result. Subsequently, WES was performed and analyzed by bioinformatics. The clinical examinations and genetic diagnostic tests were recommended by physicians based on the clinical judgment, but the final decision was made by the parents/guardians. Diagnostic results (including physiological, metabolic and genetic results) were reported to the parents/guardians. Relevant recommendations (e.g., medical management changes, dietary changes, physiotherapy/psychosocial support, follow-up assessment and reproductive planning) were proposed by physicians, but autonomous decisions were made by the parents/guardians. During the study process, the parents/guardians signed waivers of informed consent and could withdraw from the study at any time. This study was approved by the Ethics Committee of PLA General Hospital (No. S2016-120-02). Work was performed in accordance with the Declaration of Helsinki.

### WES analysis

Genomic DNA was isolated from peripheral blood of the probands and/or their biological parents. WES was performed and analyzed by (Kaiumph Medical Diagnostic Lo. Ltd, (Beijing, China) [19], Angen Gene Medicine Tech (Beijing, China) [20] or Running Gene Inc. (Beijing, China) [21] using their own bioinformatics pipelines as previously described. The laboratory-specific WES methodologic parameters were shown in Supplementary Table 1. Briefly, reads were cleaned to pass quality controls and were aligned to the reference human genome (GRCH37/hg19, genome.ucsc.edu) by BWA-MEM. SNVs and Indels were detected by GATK, and annotated by ANNOVAR (annovar.openbioinformatics.org/en/latest/). Variants were filtered using public databases (including dbSNP142, 1000 Genomes, and ESP6500, ExAc, and in-house Chinese Exome Database) [22–24], and/or published papers in WOSCC and PubMed database. Deleterious SNVs were predicted by ReVe (varcards.biols.ac.cn/); VEST3 (wiki.chasmsoftware.org/index.php/Main_Page); REVEL (sites.google.com/site/revelgenomics); and CADD (cadd.gs.washington.edu). Variants were classified using the recommended terminology “pathogenic (P)”, “likely pathogenic (LP)”, “uncertain significance (VUS)”, “likely benign (LB)”, and “benign (B)” according to the recommendation of ACMG [25]. P/LP variations were selected as causative variations for DD/ID in this study. The diagnostic yield of DD/ID was calculated as the total number of DD/ID children with P/LP variants divided by the total number of DD/ID children.

### Statistical analysis

Statistical analyses were performed using SPSS version 28.0. Descriptive statistics was performed to describe demographic and basic clinical features. Results were presented as numbers, median, percentage, and 95% confidence interval (CI). The Wilcoxon rank-sum test was used for age-group comparison. Categorical variables were presented as numbers, and a chi-square test was used for between group comparisons. The variance inflation factor (VIF) was used for the multicollinearity test and variable selection. Clinical variables found to be associated with P/LP variants in a univariate analysis (*p* < 0.2) were further included in a multivariate logistic regression analysis, and results were presented as odds ratios (ORs) and 95% CI. *P* < 0.05 was considered statistically significant.

## Results

### Demographic and basic clinical features of enrolled patients

**Fig. 2:**
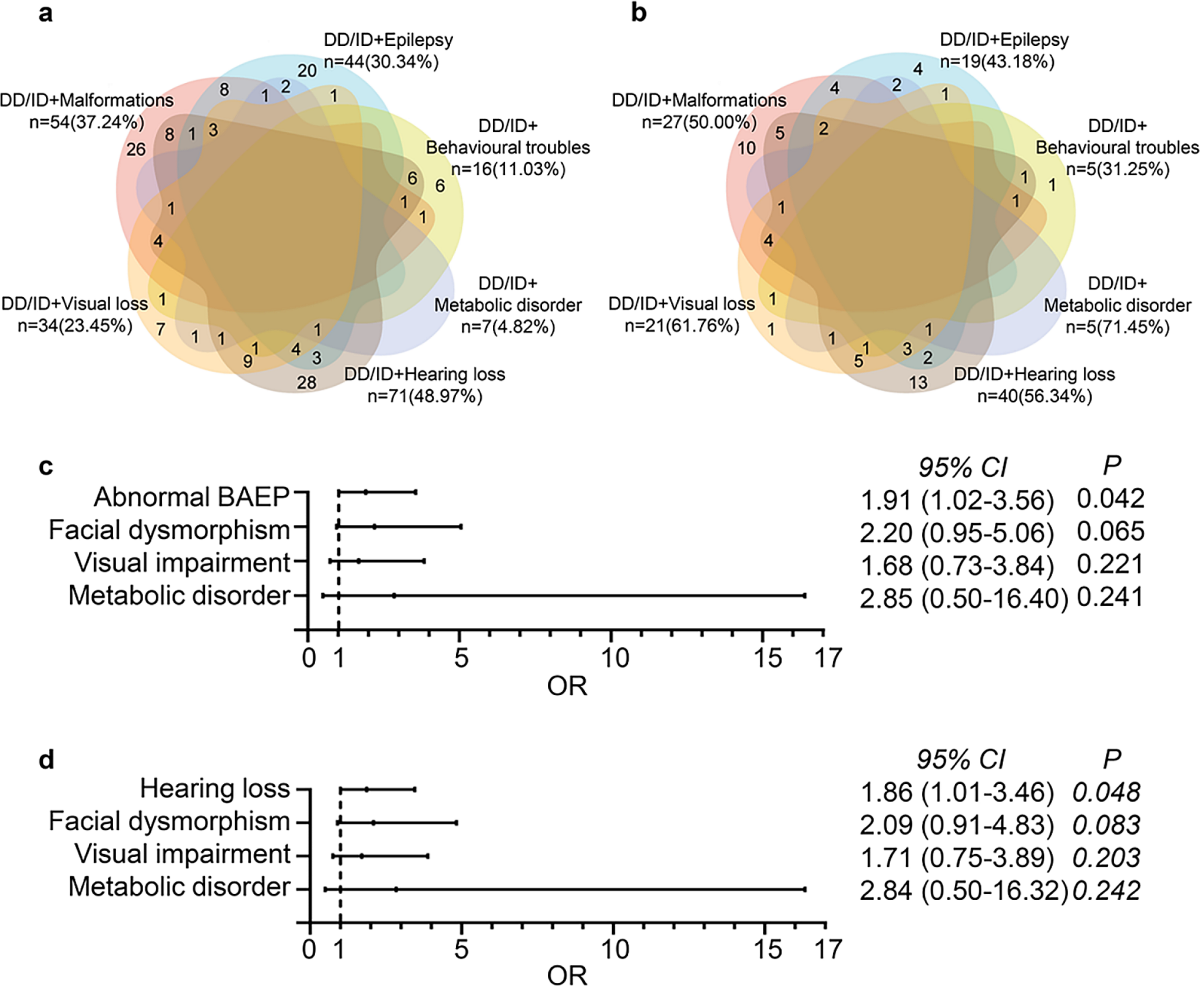
Diagnostic rate of WES. (**a**) Patient classification among six subgroups of syndromic DD/ID. (**b**) Patient with diagnostic SNV/Indels in these six subgroups of syndromic DD/ID. The six colored irregular graphics in (**a**) and (**b**) represent the six subgroups of syndromic DD/ID: brown for DD/ID with hearing loss, pink for DD/ID with malformations, blue for DD/ID with epilepsy, orange for DD/ID with visual loss, yellow for DD/ID with behavioural troubles and purple for DD/ID with metabolic disorders. Overlap between the different irregular graphics shows the overlap of patients among these subgroups. (**c**) Hearing loss, visual loss, facial dysmorphism, and metabolic disorders were analyzed in the logistic regression model. (**d**) Abnormal BAEP, visual loss, facial dysmorphism, and metabolic disorders were analyzed in the logistic regression model. DD, developmental disorder; ID, intellectual disability; BAEP, brainstem auditory evoked potential; 95% CI, 95% confidence interval; OR, odds ratio

We reviewed and analyzed the WES results of 225 patients diagnosed with DD/ID, of which 208 were trio-WES data (trio: proband and biological parents), one was duo-WES data (duo: proband and one biological parent), and another 16 was singleton-WES data (singleton: proband only, no parents were available). The study group had a median age of 2.58 years and was 64.44% male. Detailed demographic and basic clinical features are shown in Table 1. Overall, 56.21%, 55.84%, 79.31%, and 88.89% patients were found to have an abnormal brain magnetic resonance imaging (MRI), electroencephalogram (EEG), abnormal brainstem auditory evoked potential (BAEP), and visual evoked potential (VEP) results, respectively. Affected children displayed multiple clinical manifestations with 64.89% of patients displaying ID, 52.44% with speech delay, 46.22% with motor delay, 31.56% with hearing loss, 15.11% visual loss, 19.56% with seizures, and 12.44% facial dysmorphism. Based on clinical phenotypes, children were classified into two main groups: an isolated DD/ID group (*n* = 80, 35.56%) and a syndromic DD/ID group (*n* = 145, 64.44%). Furthermore, children in the latter group were further divided into six subgroups: (1) DD/ID with hearing loss (*n* = 71, 48.97%); (2) DD/ID with malformations (*n* = 54, 37.24%); (3) DD/ID with epilepsy (*n* = 44, 30.34%); (4) DD/ID with visual loss (*n* = 34, 23.45%); (5) DD/ID with behavioural troubles (*n* = 16, 11.03%); and (6) DD/ID with metabolic disorders (*n* = 7, 4.83%). Since an affected child may have multiple clinical manifestations, the same child may be classified in different subgroups. Figure 2a shows the detailed information on patient classification among six subgroups of syndromic DD/ID.

**Table 1:** Clinical characteristics of 96 patients with diagnostic results among the 225 patients tested for diagnostic whole-exome sequencing

Characteristics	Individuals, n(%)	P/LP diagnosed individuals, n(%)	*P* value
**Gender**			0.548
Male	145(64.44)	64(44.14)	
Female	80(35.56)	32(40.00)	
Total	225(100.00)	96(42.67)	
**Age(year)**			0.536
< 2	89(39.55)	37(41.57)	
(2–6)	94(41.78)	43(45.74)	
≥ 6	42(18.67)	16(38.10)	
Family history	35(15.56)	17(48.57)	0.442
**Classification**			0.548
Isolated DD/ID	80(35.56)	32(40.00)	
Sydromic DD/ID	145(64.44)	64(44.14)	
Subgroups of Sydromic DD/ID			0.221
DD/ID + Hearing loss	71(48.97)	40(56.34)	
DD/ID + Malformations	54(37.24)	27(50.00)	
DD/ID + Epilepsy	44(30.34)	19(43.18)	
DD/ID + Visual loss	34(23.45)	21(61.76)	
DD/ID + Behavioural troubles	16(11.03)	5(31.25)	
DD/ID + Metabolic disorder	7(4.83)	5(71.43)	
**Clinical examinations**			
Abnormal Brain MRI	95/169(56.21)	42(44.21)	0.9
Abnormal EEG	86/154(55.84)	35(40.70)	0.331
Abnormal BAEP	69/87(79.31)	39(56.52)	0.03
Abnormal VEP	24/27(88.89)	14(58.33)	0.569
**Clinical features**			
Intellectual disability	146/225(64.89)	60(41.10)	0.517
Speech delay	118/225(52.44)	48(40.68)	0.526
Motor delay	104/225(46.22)	47(45.19)	0.478
Hearing loss^a^	71/225(31.56)	40(56.34)	0.005
Seizures/epilepsy	44/225(19.56)	19(43.18)	0.939
Dystonia	37/225(16.44)	18(48.65)	0.421
Visual loss^b^	34/225(15.11)	21(61.76)	0.005
Social Dysfunction	31/225(13.78)	14(45.16)	0.762
Facial dysmorphism^c^	28/225(12.44)	17(60.71)	0.039
Congenital heart disease^d^	20/225(8.89)	9(45.00)	0.825
Short stature	19/225(8.44)	10(52.63)	0.359
Limb defects^e^	12/225(5.33)	5(41.67)	0.943
Congenital anomalies of Urogenital system^f^	9/225(4.00)	5(55.56)	0.501
Metabolic disorder	7/225(3.11)	5(71.43)	0.14
Stereotypic behaviors	7/225(3.11)	3(42.56)	1
Autism spectrum disorder	6/225(2.67)	1(16.67)	0.243
Dysphagia	4/225(1.78)	3(75.00)	0.315
ADHD	3/225(1.33)	1(33.34)	1
Macrocephaly	3/225(1.33)	1(33.34)	1
Microcephaly	2/225(0.89)	1(50.00)	1
Ataxia	1/225(0.44)	1(100.00)	0.427

P/LP, pathogenic or likely pathogenic; DD, developmental disorder; ID, intellectual disability; MRI, magnetic resonance imaging; EEG, electroencephalogram; ADHD, attention deficit and hyperactivity disorder; BAEP, brainstem auditory evoked potential; VEP, visual evoked potential

^a^ Assessed by BAEP, Universal Newborn Hearing Screening, Ear-nose-throat (ENT) specialists and/ or clinical questionnaire

^b^ Included abnormal VEP signal, strabismus, cortical visual impairment, hypermetropia, ptosis, nystagmus, myopia, etc

^c^ Included cleft lip and cleft palate, dysplasia of auricle, ocular hypertelorism, micrognathia, low set ears, flat nasal bridge, etc

^d^ Included Ventricular septal defect, patent ductus arteriosus, patent foramen ovale, etc

^e^ Included Foot rotation, spina bifida, scoliosis, arthrogryposis, hexadactyly, etc

^f^ Included Ectopic ureteral orifice, anal stenosis, intestinal obstruction, renal dysplasia, micropenis, hypospadias, etc

### Diagnostic rate of WES

As shown in Table 1, an overall diagnostic yield of 42.67% (96/225, 95% CI: 36.15-49.18%) was achieved, and there was no significant difference in different age groups (*P* = 0.536), gender groups (*P =* 0.548), or family history (*P =* 0.442). In the isolated DD/ID group, the yield was 40.00% (32/80, 95% CI: 29.03-50.97%), close to that of syndromic DD/ID group (64/145, 95% CI: 35.96-52.32%), with no significant difference (*P =* 0.548). And in the six phenotype subgroups of syndromic DD/ID group, there were no significant difference from one another (*P =* 0.221), although the diagnostic yield ranged from 31.25 to 71.43%. Figure 2b shows the details of children with diagnostic SNV/Indels in these six subgroups of syndromic DD/ID.

Furthermore, the diagnostic yield varied in different clinical features (Table 1), hearing loss, visual loss and facial dysmorphism can raise the diagnostic yield, and these three effects were all statistically significant (*P* = 0.005, *P* = 0.005, and *P* = 0.039, respectively). As for clinical examinations, none of these had statistical significance, except for abnormal BAEP (*P* = 0.030), which indicated that BAEP evaluation can help identify causative genetic variants in DD/ID children. Of note, children with hearing loss were mostly identified by BAEP signals (69/71), and VIF between abnormal BAEP and hearing loss was 24.61, thus, abnormal BAEP and hearing loss were separately included in a multivariate logistic regression analysis.

In the logistic regression model, hearing loss/abnormal BAEP, visual loss, facial dysmorphism, and metabolic disorders were analyzed (Fig. 2c, d). Hearing loss (OR = 1.86, 95%CI: 1.01–3.46, *P =* 0.046) or abnormal BAEP (OR = 1.91, 95%CI: 1.02–3.56, *P =* 0.042) was independently associated with causative genetic variations (Fig. 2c, d).

These findings confirmed our hypothesis that systematic clinical phenotyping of DD/ID is important for increasing the diagnostic yield of WES, and we should emphasize the great value of physiological, biochemical and genetic tests in the diagnosis of DD/ID.

### Inheritance patterns among diagnostic SNVs/Indels

**Fig. 3:**
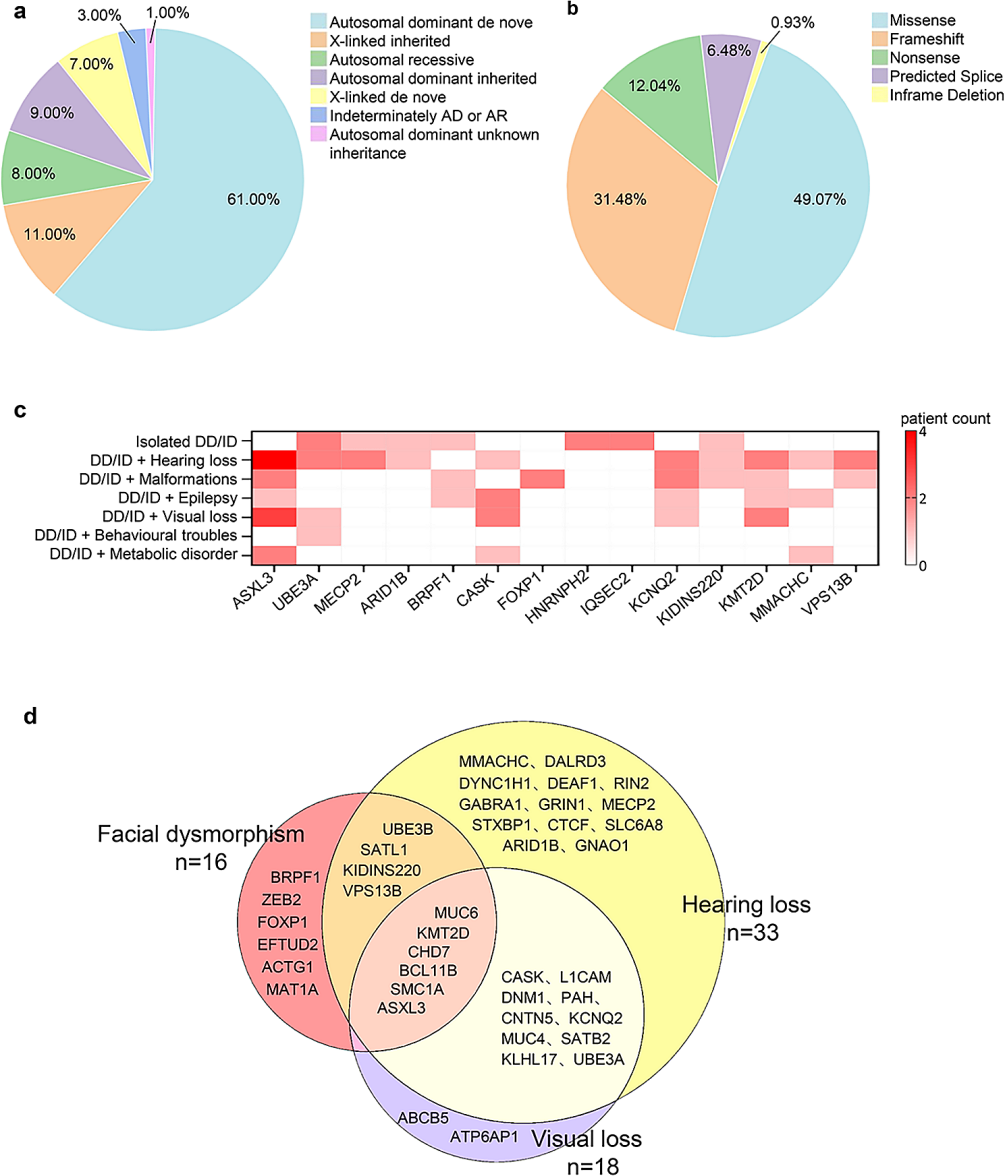
Diagnostic SNVs/Indels were identified in our cohort. (**a**) Inheritance patterns among diagnostic SNVs/Indels. (**b**) Mutation types among diagnostic SNVs/Indels. (**c**) Heatmap of identified causative genes with diagnostic SNVs/Indels among phenotype groups. Genes appeared in ≥ 2 patients are displayed. The color of each cell represents the number of patients diagnosed by the specific gene (row) in the relevant phenotype group (column). (**d**) Distribution of diagnostic genes in different clinical features. The three circles represent the three clinical features: yellow for hearing loss, purple for visual loss, and pink for facial dysmorphism. Overlap between the different circles shows the overlap of genes among these clinical features. DD, developmental disorder; ID, intellectual disability; SNVs, single nucleotide variations; Indels, small insertions/deletions

In the cohort, 108 diagnostic SNVs/Indels were found in 96 patients with variants spanning 81 genes. The detailed diagnostic SNVs/Indels are shown in Supplementary Table 2. The inheritance patterns in 100 conditions (96 cases, two cases with two conditions caused by P/LP variants in different genes, one case with three conditions caused by P/LP variants in different genes) were identified, including 61.00% were autosomal dominant and de novo, 11.00% were X-linked and inherited, 9.00% were autosomal dominant and inherited, 8.00% were autosomal recessive, 7.00% were X-linked and de novo, 3.00% were indeterminately autosomal dominant or recessive, and 1.00% were autosomal dominant of unknown origin due to lack of parental samples (Fig. 3a).

### Mutation types among the 108 diagnostic SNVs/Indels

Among the 108 diagnostic SNVs/Indels, 53 (49.07%) were missense variants, 34 (31.48%) were frameshift variants, 13 (12.04%) were stop-gained variants, 7 (6.48%) were predicted splice variants, and 1 (0.93%) was inframe deletion (Fig. 3b). Half of the 108 mutant alleles were truncating (nonsense, splicing, or frameshift) and others were nontruncating (missense or in-frame deletions).

### Genes with diagnostic variants within multiple patients

Mutations were identified in 81 different genes, 14 (17.28%) of which were identified in two or more patients (Supplementary Table 2). Mutations in two genes (*ASXL3* and *UBE3A*) were each found in four patients, mutations in *MECP2* were found in three patients, and mutations in 11 genes (*MMACHC*, *VPS13B*, *ARID1B*, *BRPF1*, *CASK*, *FOXP1*, *HNRNPH2*, *IQSEC2*, *KCNQ2*, *KIDINS220*, and *KMT2D*) were each found in two patients (Supplementary Table 2). Furthermore, among these 14 genes, seven genes influenced the isolated DD/ID group, *HNRNPH2* and *IQSEC2* were only detected in this group (Fig. 3c). Meanwhile, *FOXP1* was only detected in DD/ID with malformation group, and *ASXL3* was most frequently involved in DD/ID with hearing loss group (Fig. 3c).

Figure 3d shows the diagnostic genes identified in patients with hearing loss, visual loss and/or facial dysmorphism. Forty-one genes were involved, and six genes: *MUC6*, *KMT2D*, *CHD7*, *BCL11B*, *SMC1A*, and *ASXL3*, were detected in all three clinical manifestations (Fig. 3d).

### Multiple gene findings in one patient

Although cases with multiple genetic diagnoses are rare, in this study, two cases revealed two genetic diagnoses, and one case had three distinct genetic diagnoses (Supplementary Table 3). Both (or all three) gene findings can explain the major or all nonoverlapping/overlapping clinical features. For example, one patient with DD and hypotonia had a de novo pathogenic variant in three genes: *PRDM16*, *SETD2*, and *KRT9.* One patient with epilepsy and ID had a de novo missense pathogenic variant in *EIF4G1* and *HSPB1*.

### Impact of WES on medical management

Supplementary Table 4 showed that WES results changed medical management and impacted family planning for DD/ID children (45/96), including reproductive decision changes (*n* = 22), initiation of disease monitoring/systemic investigation (*n* = 5), discontinuation medication (*n* = 6), addition of medication (*n* = 30), physiotherapy/psychosocial support (*n* = 12), and ending a diagnostic “odyssey” (*n* = 6). For example, a compound heterozygous pathogenic variant in MMACHC was detected in a child (Patient 210) with DD, seizures, methylmalonic acidaemia and homocysteinaemia that was treatable with vitamin B12, betaine, folate, and levetiracetam. Hypermethioninemia due to MAT1A mutation was diagnosed in a child (Patient 156) with DD, who was subsequently treated with a methionine-restricted diet in combination with rehabilitation treatment after diagnosis and experienced significant growth improvement. A child with DD (Patient 119) who harboured anSTXBP1 variant had been empirically started on phenobarbital to stop the seizures but showed no response; after molecular diagnosis, levetiracetam was added to the treatment protocol, which was successful, and the patient was seizure free for years. In summary, the data emphasize the significant implications of genetic diagnosis established by WES for patients and their families.

## Discussion

In this study, WES was performed for 225 Chinese children with DD/ID, an overall diagnostic yield of 42.67% was achieved. 108 diagnostic SNVs/Indels in 81 genes were found in the cohort, most of which were de novo and protein altering. Hearing loss, visual loss, and facial dysmorphism had significant effect on diagnostic yield. Of note, hearing loss or abnormal BAEP was more likely to have causative genetic mutations.

In heterogeneous populations, the diagnostic yields of DD/ID with WES vary widely depending on the sample size of the study [26–28]. In a study of 38 patients with ID and microcephaly, a positive diagnosis was revealed by WES in 29% (11/38) [27]. In a cohort of 232 children with DD/ID, WES identified P/LP SNVs in 39% of the patients (91/232) [28]. In our paediatric DD/ID cohort, the diagnostic yield of WES was relatively high at 42.67%, which was partly due to the following reasons: (1) There was a high rate of trio sequencing in our cohort (208/225, 92.44%), which numerous studies have proven that trio sequencing can increase the diagnostic yield [29]. (2) Subjects in our cohort may exhibit selection bias. Our paediatric clinic is one of the top paediatric clinics in China, and the most severely affected children are referred to the top clinics for diagnosis and management. Thus, the children seen in our clinic had a relatively high rate of dysmorphism and/or multiple organ system abnormalities. Given that specific clinical features can increase the diagnostic yield of WES [4, 16], the comparatively severe clinical features of the children in our study may have increased the diagnostic yield of WES; furthermore, these children had undergone extensive prior routine physiological and biochemical testing, and WES was conducted due to a high suspicion that the child had a genetic disorder [15].

In this study, we found that the diagnostic yields for isolated and syndromic DD/ID were equivalent, which was consistent with previous reports [1, 4, 16, 17]. Dong XR et al. found that although there was no significant difference between isolated and syndromic DD, there were significant differences among the four subgroups of syndromic DD. The diagnostic rate of DD in the behavioural troubles subgroup was significantly lower than that in the other three subgroups (i.e., DD with malformation, DD with epilepsy, and DD with metabolic disorder), and these three subgroups were not significantly different from one another [1]. Conversely, in our study, we found that there were no significant differences among the six subgroups of syndromic DD/ID, which can be partially attributed to the smaller sample sizes of some subgroups, such as the DD/ID with behavioural troubles group (*n* = 16), and the DD/ID with metabolic disorder group (*n* = 7), which may have resulted in underpowered statistical tests. It is worth noting that although the numbers of patients in our study with some specific clinical features were still modest, our study demonstrated a diagnostic yield of at least 30% for these clinical features (Table 1), which supported the powerful and valuable effects of WES in identifying the genetic aetiology of DD/ID. Furthermore, we found that hearing loss, visual loss, and facial dysmorphism significantly increased the diagnostic yield of WES in patients with DD/ID; notably, hearing loss and abnormal BAEP were independently associated with causative genetic variations, which further confirmed that specific clinical features can significantly increase genetic diagnostic yields in DD/ID and emphasized the importance of routine physiological tests in genetic aetiology analysis in DD/ID patients.

Hearing loss is one of the common specific impairments that were modeled as sequelae of specific health disorders of children [12, 30, 31], and it is also a common clinical feature in DD/ID patients [31–33]. And early detection of hearing loss is vital to language development [34, 35]. But few studies have tested the relationship between hearing loss and the diagnostic rate of DD/ID. Hearing evaluation through subjective tests is difficult in young and uncooperative children, BAEP is reliable and effective tool in this setting [35–37]. Lau WL et al. had found the prevalence of hearing deficit in children with Down syndrome in Hong Kong was 36% (18/55) measured by BAEP [35]. BAEP is also used to assess neuronal maturation [37, 38]. In our study, 69 children (97.18%) were identified having hearing loss by BAEP and most children had a mild bilateral lesion (Supplementary Table 5). Furthermore, abnormal BAEP was independently associated with causative genetic variations, which suggested BAEP screen should be encouraged in DD/ID children.

There were 39 genes were identified in DD/ID children with abnormal BAEP in the cohort (Supplementary Table 6), most genes were associated with neurodevelopmental disorders such as DD, ID, epilepsy, and ear/hearing anomalies. And their biological processes are mainly related with nervous system development, positive regulation of cellular biosynthetic process, generation of neurons, brain development, neurogenesis, single-multicellular organism process, sensory perception of sound, inner ear morphogenesis (Supplementary Table 6). These findings suggested a need for detailed research on these genes in future.

Importantly, positive genetic results can not only end a diagnostic “odyssey”, but also beneficially influence medical care and reproductive decision [39–41], which were also observed in our studies (Supplementary Table 4). A random-effects meta-analysis showed that genetic results changed clinical management (range: 2–49%, *n* = 6 studies) and impacted reproductive planning (range: 42–100%, *n* = 4 studies) for patients with neurodevelopmental disorders (ID/DD, and/or ASD) [18]. These data strongly indicate the extremely beneficial of WES in early diagnose and personalized treatment of DD/ID, as well as in genetic counseling for DD/ID patients and families.

Although WES should be considered in the early stage of the diagnosis process, physicians should not ignore the importance of routine physiological and biochemical tests, since these examinations often substantiate the genetic testing results. In our study, specific clinical features (such as hearing loss, visual loss, and facial dysmorphism) significantly increased the diagnostic yield of WES in patients with DD/ID. Moreover, in our study, we found that hearing loss and abnormal BAEP were more likely to have causative genetic mutations. Given that early diagnosis of hearing loss and hearing rehabilitation promote language, academic and social development [42–44], hearing impairment/BAEP tests should be conducted as part of newborn screening, as well as in evaluations of DD/ID children. Early intervention and treatment based on physiological, metabolic and genetic findings can lead to better prognoses, even preventing the development of DD/ID. For example, in a study of 149 Chinese patient with cobalamin C deficiency harbouring the MMACHC c.609G > A homologous mutation, 1.3% (2/149) were prenatally diagnosed with metabolic and genetic tests, treated after birth and showed normal development; 10.1% (15/149) were diagnosed by newborn screening(10 children were treated at 15 days of age and showed normal development, while the other five children were treated after onset and all developed severe DD because of poor treatment compliance); and 88.6% (132/149) were diagnosed after onset and received personalized treatment, with various neurological sequelae (including DD, seizure, etc.) observed although most patients improved [45]. Consistent with these results, in our study, we identified a genetic aetiology in two DD/ID children (Patient 210, Patient 156) by metabolic and WES tests, and early intervention/treatment was applied; one patient showed normal development and the other patient showed significant improvement. Taken together, these data strongly indicate that physiological, metabolic, and genetic screening and early personalized treatment are pivotal for preventing DD/ID.

With the advancement of genomic technology, genetic findings in research concerning individual health are an ethical challenge and concern [15]: (1) Since children cannot legally sign informed consent on their own behalf, the parents/guardians ultimately made the final decision in this cohort, which was consistent with other studies [46]. Ross LF et al. reported that hindering children’s involvement increased the risk that medical professionals and parents would lose the children’s trust if they believed that they had no right to express their feelings and suggested that minors should be able to make informed decisions regarding their genomic evaluation [47]. (2) In this study, laboratories classified variants as P, LP, and/or VUS. After reviewing the data, we returned all genomic results to the parents/guardians, including “incidental” or “secondary” findings, which were unrelated to the reason for ordering WES but may have future medical implications [15]. Notably, we focused more on medically “actionable” findings (e.g., the availability of relevant targeted therapies or relevant risk reduction interventions) and avoided overinterpreting the clinical significance of VUSs. (3) We were bound to provide recommendations, but we respected the parents’/guardians’ beliefs, feelings, religion, and cultural traditions; autonomous decisions (e.g. therapy, reproductive planning, and follow-up assessment.) should be made by parents/guardians, although genetic results may have the potential to improve a patient’s health through effective medical intervention, or to impact family planning [48–50]. (4) Although we encouraged more family members to participate in WES testing to explore genetic aetiology [25, 48], we did not disclose the WES results to other family members without permission.

This the study has several limitations: (1) WES will not reliably detect large deletion/insertion, translocation/transversion, mitochondrial DNA, epigenic variants, or nonexonic regulatory regions, which could be caused DD/ID [4, 15, 51]. (2) There was potential selection bias for this single-center study. Some children with DD/ID did not been recruited in this cohort for various reasons (e.g. parents/guardians refused to perform WES or refused to sign informed consents, incomplete medical records, etc.). (3) WES was performed through three different WES laboratories that use their own bioinformatics pipelines [15, 16]. However, these three laboratories used the same variant-level classification according to the recommendations of the ACMG; there were no significant differences in the diagnostic yields among them (Supplementary Table 7); and all genetic results were reviewed by the ordering physicians. (4) The clinical phenotype of a child may be the result of interactions of different genes and/or environmental factors [15]. (5) Although clinical management can be guided by genetic results, cases with significantly improved effectiveness were still relatively rare (*n* = 12) compared with the number of DD/ID children (*n* = 225) (Supplementary Table 4). There are large gaps in the current knowledge on personalized genomic treatment in DD/ID patients, which underscores the importance of collaboration between genetic researchers and clinical physicians (including paediatricians, paediatric rehabilitation specialists, and paediatric neurologists) to accelerate basic and clinical research.

## Conclusion

In conclusion, our study identified genetic etiology in 42.67% of patients with DD/ID in Beijing, China, which supported that the powerful and valuable effects of WES in identifying the genetic etiology of DD/ID. Given that abnormal BAEP is independently associated with causative genetic variations, there is a need for the development of BAEP screen in DD/ID children. Despite present limitations, WES still serves as a critical tool in pediatric neurology practices.

### Electronic supplementary material

Below is the link to the electronic supplementary material.

Supplementary Material 1

Supplementary Material 2

Supplementary Material 3

Supplementary Material 4

Supplementary Material 5

Supplementary Material 6

Supplementary Material 7

## Data Availability

These sequence data have been submitted to the NCBI Sequence Read Archive (SRA) under accession number: PRJNA1067565 (https://www.ncbi.nlm.nih.gov/bioproject/PRJNA1067565).

## Abbreviations

DD: Developmental Delay
ID: Intellectual Disability
WES: Whole-exome Sequencing
SNVs: Single Nucleotide Variants
BAEP: Brainstem Auditory Evoked Potential
ACMG: American College of Medical Genetics and Genomics
DNA: Deoxyribonucleic Acid
P: Pathogenic
LP: Likely Pathogenic
VUS: Uncertain Significance
LB: Likely Benign
B: Benign
CI: Confidence Interval
VIF: Variance inflation factor
ORs: Odds Ratios
MRI: Magnetic Resonance Imaging
EEG: Electroencephalogram
VEP: Visual Evoked Potential
