# Supplementary material for: Genetic and phenotypic analysis of 225 Chinese children with developmental delay and/or intellectual disability using whole-exome sequencing
Source: BMC Genomics. 2024 Apr 22;25:391. doi: 10.1186/s12864-024-10279-1 (PMC11034079; doi:10.1186/s12864-024-10279-1)
Supplement: Supplementary file 1 — Supplementary Material 1 [file 12864_2024_10279_MOESM1_ESM.docx]

**Supplementary Table 1** Laboratory-specific WES methodologic parameters

| WES laboratories | Average depth | Average coverage | Sequencing platform |
| --- | --- | --- | --- |
| AGMT ^a^ | 100X | 99% | Illumina NovaSeq 6000 |
| KMD ^b^ | 100X | 99% | Illumina HiSeq 2000 |
| RGI ^c^ | 100X | 99% | Illumina HiSeq 2000 |

^a^ Angen Gene Medicine Tech (Beijing, China)

^b^ Kaiumph Medical Diagnostic Lo. Ltd (Beijing, China)

^c^ Running Gene Inc. (Beijing, China)
