# Supplementary material for: Genetic and phenotypic analysis of 225 Chinese children with developmental delay and/or intellectual disability using whole-exome sequencing
Source: BMC Genomics. 2024 Apr 22;25:391. doi: 10.1186/s12864-024-10279-1 (PMC11034079; doi:10.1186/s12864-024-10279-1)
Supplement: Supplementary file 2 — Supplementary Material 2 [file 12864_2024_10279_MOESM2_ESM.docx]

**Supplementary Table 2** Diagnostic SNVs/Indels identified in the cohort

| Gene | Patient Num | Transcript | Nucleotide substitution | Inheritance pattern | Type | Zygosity |
| --- | --- | --- | --- | --- | --- | --- |
| ASXL3 | Patient 010 | NM_030632 | c.3872_3875del, p.Cys1292fs*0 | AD, De nove | Frameshift | Compoud Het |
|  | Patient 033 | NM_030632 | c.2035G>T, p.Glu679X | AD, Inherited | Nonsense | Compoud Het |
|  | Patient 214 | NM_030632 | c.4120_4123dupATAG, p.A1375Dfs*7 | AD, De nove | Frameshift | Compoud Het |
|  | Patient 223 | NM_030632.1 | c.3329_3353dupTCTTTGCAAAGCATCAAGCTCGAGC, p.H1119Lfs*15 | AD, De nove | Frameshift | Compoud Het |
| UBE3A | Patient 036 | NM_130839 | c.1314dupA, p.Pro439Thrfs*6 | AD, Inherited | Frameshift | Compoud Het |
|  | Patient 037 | NM_130839 | c.1314dupA, p.Pro439Thrfs*6 | AD, Inherited | Frameshift | Compoud Het |
|  | Patient 181 | NM_001354509 | c.1309dupA, p.M437Nfs*3 | AD, Inherited | Frameshift | Compoud Het |
|  | Patient 182 | NM_001354509 | c.1309dupA, p.M437Nfs*3 | AD, Inherited | Frameshift | Compoud Het |
| MMACHC | Patient 076 | NM_015506 | c.80A>G, p.Gln27Arg;  c.609G>A, p.Trp203X | AR, Inherited | Missense/  Nonsense | Compoud Het |
|  | Patient 210 | NM_015506 | c.394C>T, p.R132*;  c.658_660delAAG, p.K220del | AR, Inherited | Nonsense/  Inframe Deletion | Compoud Het |
| VPS13B | Patient 028 | NM_017890 | c.1492_1495del, p.Phe499Ilefs*27; c.291+1G>A, splice | AR, Inherited | Frameshift/  Predicted Splice | Compoud Het |
|  | Patient 029 | NM_017890 | c.1492_1495del, p.Phe499Ilefs*27; c.291+1G>A, splice | AR, Inherited | Frameshift/  Predicted Splice | Compoud Het |
| MECP2 | Patient 038 | NM_004992 | c.1138_1172del, p.Val380fs*0 | XLD/XLR, Inherited | Frameshift | Compoud Het |
|  | Patient 039 | NM_004992 | c.1138_1172del, p.Val380fs*0 | XLD/XLR, Inherited | Frameshift | Hemi |
|  | Patient 080 | NM_001110792 | c. C952T, p.R318C | XLD, De nove | Missense | Compoud Het |
| ARID1B | Patient 125 | NM_001346813 | c.4587delT, p.Y1530Tfs*3 | AD, De nove | Frameshift | Compoud Het |
|  | Patient 131 | NM_020732 | c.2920_2926del, p.A974fs | AD, De nove | Frameshift | Compoud Het |
| BRPF1 | Patient 012 | NM_004634 | c.A3446C, p.D1149A | AD, De nove | Missense | Compoud Het |
|  | Patient 175 | NM_001003694 | c.2041dupT, p.D680fs | AD, De nove | Frameshift | Compoud Het |
| CASK | Patient 047 | NM_003688 | c.G62A, p.G21D | XLD/XLR, Inherited | Missense | Hemi |
|  | Patient 048 | NM_003688 | c.G62A, p.G21D | XLD/XLR, Inherited | Missense | Hemi |
| FOXP1 | Patient 062 | NM_001349338 | c.447_450del, p.Lys149Asnfs*55 | AD, De nove | Frameshift | Compoud Het |
|  | Patient 197 | NM_032682.5 | c.1420A>T, p.I474F | AD, De nove | Missense | Compoud Het |
| HNRNPH2 | Patient 132 | NM_001032393 | c.616C>T, p.R206W | XLD, De nove | Missense | Compoud Het |
|  | Patient 179 | NM_001032393 | c.616C>T, p.R206W | XLD, De nove | Missense | Compoud Het |
| IQSEC2 | Patient 115 | NM_001111125 | c.2563C>T, p.R855X | XLD, Inherited | Nonsense | Compoud Het |
|  | Patient 116 | NM_001111125 | c.2563C>T, p.R855X | XLD, Inherited | Nonsense | Compoud Het |
| KCNQ2 | Patient 079 | NM_172107 | c.1678C>T, p.Arg56 | AD, De nove | Missense | Compoud Het |
|  | Patient 195 | NM_172107 | c.1087T>G, p.Y363D | AD, De nove | Missense | Compoud Het |
| KIDINS220 | Patient 092 | NM_001348729 | c.4341_4344del, p.Arg14 | AD, De nove | Frameshift | Compoud Het |
|  | Patient 097 | NM_001348729 | c.4281_4282insCT, p.Ile1428Leufs*5 | AD, De nove | Frameshift | Compoud Het |
| KMT2D | Patient 100 | NM_003482 | c.16200C>G, p.Tyr5400X | AD, De nove | Nonsense | Compoud Het |
|  | Patient 207 | NM_003482.3 | c.8743C>T, p.R2915* | AD, De nove | Nonsense | Compoud Het |
| B3GALNT2 | Patient 150 | NM_152490 | c.979G>A, p.D327N；  c.657_658insTT, p.E220fs | AR, Inherited | Missense/  Frameshift | Compoud Het |
| PAH | Patient 142 | NM_000277 | c.532G>A, p.E178K;  c.350C>T, p.T117I | AR, Inherited | Missense/  Missense | Compoud Het |
| TH | Patient 133 | NM_000360 | c.605G>A, p.R202H;  c.364C>T, p.R122X | AR, Inherited | Missense/  Missense | Compoud Het |
| UBE3B | Patient 134 | NM_130466 | c.3135C>G, p.Y1045X;  c.3164T>C, p.L1055P | AR, Inherited | Missense/  Missense | Compoud Het |
| ABCB5 | Patient 014 | NM_001163941 | c.1750T>C, p.Ser584Pro | AD, De nove | Missense | Compoud Het |
| ACTA2 | Patient 201 | NM_001613.2 | c.535C>T, p.R179C | AD, De nove | Missense | Compoud Het |
| ACTG1 | Patient 208 | NM_001614 | c.335C>T, p.P112L | AD, De nove | Missense | Compoud Het |
| AHDC1 | Patient 032 | NM_001029882 | c.1313delC, p.Pro438Leufs*13 | AD, De nove | Frameshift | Compoud Het |
| ATP1A3 | Patient 224 | NM_152296 | c.2408G>A, p.G803D | AD, De nove | Missense | Compoud Het |
| ATP6AP1 | Patient 104 | NM_001183 | c.43C>T, p.Arg15X | XLR, Inherited | Nonsense | Hemi |
| BCL11B | Patient 008 | NM_138576 | c.1365C>G, p.Tyr455X | AD, De nove | Nonsense | Compoud Het |
| CDKL5 | Patient 067 | NM_001037343 | c.C2854T, p.R952X | XLD, Inherited | Nonsense | Hemi |
| CHD2 | Patient 017 | NM_001271 | c.C4318T, p.R1440X | AD, De nove | Nonsense | Compoud Het |
| CHD7 | Patient 148 | NM_017780 | c.5512_5513del, p.M1838fs | AD, De nove | Frameshift | Compoud Het |
| CLCN4 | Patient 225 | NM_001830 | c.1400G>C, p.Gly467Ala | XLD, De nove | Missense | Compoud Het |
| CNTN5 | Patient 013 | NM_014361 | c.1559G>C, p.Arg520Thr | AD, De nove | Missense | Compoud Het |
| COL4A5 | Patient 043 | NM_000495 | c.262C>T, p.Pro88Ser | XLD, Inherited | Missense | Hemi |
| CTCF | Patient 117 | NM_006565 | c.1435C>T, p.R479C | AD, De nove | Missense | Compoud Het |
| CUX2 | Patient 169 | NM_015267 | c.1768G>A, p.E590K | AD, De nove | Missense | Compoud Het |
| DALRD3 | Patient 082 | NM_018114 | c.1055A>C, p.Gln352Pro | AD, De nove | Missense | Compoud Het |
| DEAF1 | Patient 091 | NM_021008 | c.767T>G, p.Ile256Ser | AD/AR, De nove | Missense | Compoud Het |
| DNM1 | Patient 153 | NM_001005336 | c.1601C>G, p.I533M | AD, De nove | Missense | Compoud Het |
| DYNC1H1 | Patient 114 | NM_001376 | c.10353_10354del, p.A3452Rfs*32 | AD, De nove | Frameshift | Compoud Het |
| EEF1A2 | Patient 141 | NM_001958 | c.208G>A, p.G70S | AD, De nove | Missense | Compoud Het |
| EFTUD2 | Patient 152 | NM_004247 | c.257_260del, p.T86fs | AD, De nove | Frameshift | Compoud Het |
| EIF4G1 | Patient 068 | NM_182917 | c.3616C>T, p.Arg12 | AD, De nove | Missense | Compoud Het |
| FGD1 | Patient 171 | NM_004463 | c.1966C>T, p.R656X | XLR, Inherited | Missense | Hemi |
| GABRA1 | Patient 110 | NM_001127648 | c.799C>A, p.L267I | AD, De nove | Missense | Compoud Het |
| GABRB3 | Patient 151 | NM_001191320 | c.656A>C, p.K219T | AD, NA | Missense | Compoud Het |
| GLI2 | Patient 021 | NM_005270 | c.571G>A, p.Gly191Arg | AD, De nove | Missense | Compoud Het |
| GLI3 | Patient 107 | NM_000168 | c.3598C>G, p.His1200Asp | AD, Inherited | Missense | Hom |
| GNAO1 | Patient 200 | NM_020988.2 | c.724-8G>A, splice | AD, De nove | Predicted Splice | Compoud Het |
| GRIN1 | Patient 040 | NM_007327 | c.1852G>C, p.Gly618Arg | AD/AR, De nove | Missense | Compoud Het |
| GRIN2A | Patient 024 | NM_001134407 | c.2246C>T, p.Thr749Ile | AD, De nove | Missense | Compoud Het |
| GRIN2B | Patient 222 | NM_000834.3 | c.1011-1G>C, splice-3 | AD, De nove | Predicted Splice | Compoud Het |
| HDAC4 | Patient 069 | NM_006037 | c.2573_2583del, p.Leu858Profs*16 | AD, De nove | Frameshift | Compoud Het |
| HSPB1 | Patient 068 | NM_001540 | c.121G>A, p.Glu41Lys | AD, De nove | Missense | Compoud Het |
| KLHL17 | Patient 007 | NM_198317 | c.701C>T, p.Pro234Leu | AD, De nove | Missense | Compoud Het |
| KRIT1 | Patient 004 | NM_004912 | c.151_154del, p.Lys51Phefs*12 | AD, Inherited | Frameshift | Compoud Het |
| KRT9 | Patient 187 | NM_000226 | c.162_163del, p.G54fs | AD, De nove | Frameshift | Compoud Het |
| L1CAM | Patient 198 | NM_000425.3 | c.400+5G>A, splice | XLR, De nove | Predicted Splice | Hemi |
| MAT1A | Patient 156 | NM_000429 | c.494C>G, p.A165G | AD/AR, Inherited | Missense | Compoud Het |
| MEF2C | Patient 176 | NM_001131005 | c.631+2T>C, splice | AD, De nove | Predicted Splice | Compoud Het |
| MUC4 | Patient 089 | NM_018406 | c.9213_9217del, p.Pro3072Serfs*27 | AD, De nove | Frameshift | Compoud Het |
| MUC6 | Patient 158 | NM_005961 | c.C4475A, p.A1492D | AD, De nove | Missense | Compoud Het |
| NESTIN | Patient 118 | NM_006617 | c.3307_3308del, p.Q1103Gfs*37 | AD, De nove | Frameshift | Compoud Het |
| NKX2-1 | Patient 071 | NM_003317 | c.610C>T, p.Gln204X | AD, De nove | Nonsense | Compoud Het |
| NOTCH1 | Patient 159 | NM_017617 | c.5048G>C, p.R1683P | AD, De nove | Missense | Compoud Het |
| P4HTM | Patient 063 | NM_177938 | c.1585G>A, p.Ala529Thr | AD, De nove | Missense | Compoud Het |
| PRDM16 | Patient 187 | NM_022114 | c.1701_1702insGGCACGAC, p.Q567fs | AD, De nove | Frameshift | Compoud Het |
| PTEN | Patient 129 | NM_000314 | c.737C>T, p.P246L | AD, De nove | Missense | Compoud Het |
| RIN2 | Patient 124 | NM_001242581 | c.1075_1076del, p.R359Afs*6 | AD, De nove | Frameshift | Compoud Het |
| SATB2 | Patient 142 | NM_015265 | c.141dupC, p.N48fs | AD, De nove | Frameshift | Compoud Het |
| SATL1 | Patient 081 | NM_001012980 | c.907C>T, p.Gln303X | XLD, Inherited | Nonsense | Hemi |
| SCN9A | Patient 137 | NM_002977 | c.258+1G>A, splice | AD, Inherited | Predicted Splice | Compoud Het |
| SETD2 | Patient 187 | NM_014159 | c.5882A>T, p.E1961V | AD, De nove | Missense | Compoud Het |
| SLC6A8 | Patient 015 | NM_005629 | c.626_627del, p.Pro209Argfs*86 | XLR, De nove | Frameshift | Hemi |
| SMARCA2 | Patient 106 | NM_003070 | c.1490G>A, p.Arg497Gln | AD, De nove | Missense | Compoud Het |
| SMARCA4 | Patient 031 | NM_001128844 | c.3607C>T, p.Arg1203Cys | AD, De nove | Missense | Compoud Het |
| SMC1A | Patient 154 | NM_001281463 | c.800C>G, p.S267X | XLD, De nove | Missense | Compoud Het |
| SON | Patient 221 | NM_032195.1 | c.5753_5756delTTAG, p.V1918Efs*87 | AD, De nove | Frameshift | Compoud Het |
| STXBP1 | Patient 119 | NM_003165 | c.1216C>T, p.R406C | AD, De nove | Missense | Compoud Het |
| SYNGAP1 | Patient 220 | NM_006772 | c.1167_1168delAG, p.G391Qfs*27 | AD, De nove | Frameshift | Compoud Het |
| TBX6 | Patient 180 | NM_004608 | c.434C>T, p.P145L | AD, Inherited | Missense | Compoud Het |
| YAP1 | Patient 162 | NM_001130145 | c.680C>T, p.S227L | AD, De nove | Missense | Compoud Het |
| ZEB2 | Patient 147 | NM_014795 | c.1314delC, p.H438fs | AD, De nove | Frameshift | Compoud Het |
| ZNF597 | Patient 042 | NM_152457 | c.T368C, p.I123T | AD, De nove | Missense | Compoud Het |
