# Supplementary material for: Genetic and phenotypic analysis of 225 Chinese children with developmental delay and/or intellectual disability using whole-exome sequencing
Source: BMC Genomics. 2024 Apr 22;25:391. doi: 10.1186/s12864-024-10279-1 (PMC11034079; doi:10.1186/s12864-024-10279-1)
Supplement: Supplementary file 3 — Supplementary Material 3 [file 12864_2024_10279_MOESM3_ESM.docx]

**Supplementary Table 3** Patients with multiple molecular diagnoses among underlying gene findings

| Patient | Inheritance | Gene | OMIM_Disease | Mutation Origin | Mutation | Type of Mutation |
| --- | --- | --- | --- | --- | --- | --- |
| Patient 068 | AD | EIF4G1 | Parkinson disease 18 | De nove | p.Arg1206Cys | Missense |
|  | AD | HSPB1 | Charcot-Marie-Tooth disease, axonal, type 2F; Neuronopathy, distal hereditary motor, type IIB | De nove | p.Glu41Lys | Missense |
| Patient 142 | AD | SATB2 | Glass syndrome | De nove | p.N48fs | Frameshift |
|  | AR | PAH | Hyperphenylalaninemia, non-PKU mild; Phenylketonuria | Inherited | p.E178K&p.T117I | Missense/Missense |
| Patient 187 | AD | PRDM16 | Cardiomyopathy, dilated, 1LL; Left ventricular noncompaction 8 | De nove | p.Q567fs | Frameshift |
|  | AD | SETD2 | Intellectual developmental disorder,  Autosomal dominant 70; Luscan-Lumish syndrome; Rabin-Pappas syndrome | De nove | p.E1961V | Missense |
|  | AD | KRT9 | Palmoplantar keratoderma, epidermolytic, 1 | De nove | p.G54fs | Frameshift |
