# Supplementary material for: Genetic and phenotypic analysis of 225 Chinese children with developmental delay and/or intellectual disability using whole-exome sequencing
Source: BMC Genomics. 2024 Apr 22;25:391. doi: 10.1186/s12864-024-10279-1 (PMC11034079; doi:10.1186/s12864-024-10279-1)
Supplement: Supplementary file 4 — Supplementary Material 4 [file 12864_2024_10279_MOESM4_ESM.docx]

**Supplementary Table 5** BAEP results in DD/ID children

| BAER results | Individuals, n(%) |
| --- | --- |
| Normal (<20 dBnHL ) | 18(20.69) |
| Hearing loss | 69(79.31) |
| Mild (20–35 dBnHL) | 61(70.11) |
| Moderate (35–50 dBnHL) | 0 |
| Moderate–severe (50–65 dBnHL ) | 2(2.30) |
| Severe (65–80 dBnHL) | 0 |
| Profound (80-95 dBnHL) | 4(4.60) |
| Complete hearing loss (≥95 dBnHL) | 2(2.30) |
| Total | 87(100.00） |
| Bilateral | 62(89.86) |
| Unilateral | 7(10.14) |
| Left | 1(1.45) |
| Right | 6(8.70) |
| Total | 69(100.00) |

DD, developmental disorder; ID, intellectual disability; BAEP, brainstem auditory evoked potential; BAER, brainstem auditory evoked response.
