# Supplementary material for: Genetic and phenotypic analysis of 225 Chinese children with developmental delay and/or intellectual disability using whole-exome sequencing
Source: BMC Genomics. 2024 Apr 22;25:391. doi: 10.1186/s12864-024-10279-1 (PMC11034079; doi:10.1186/s12864-024-10279-1)
Supplement: Supplementary file 5 — Supplementary Material 5 [file 12864_2024_10279_MOESM5_ESM.docx]

**Supplementary table 6** Genes identified in DD/ID with BAEP group

| Gene | OMIM_Disease | Biological process |
| --- | --- | --- |
| ASXL3 | Bainbridge-Ropers syndrome, (615485) | regulation of transcription, DNA-templated, animal organ morphogenesis, positive regulation of transcription from RNA polymerase II promoter, negative regulation of lipid biosynthetic process. |
| VPS13B | Cohen syndrome, (216550) | acrosome assembly, lipid transport, Golgi organization, nervous system development, central nervous system development, protein transport, vesicle-mediated transport, neuron projection development, slow endocytic recycling, maintenance of lens transparency, adipose tissue development. |
| KCNQ2 | Myokymia, (121200);Seizures, Benign neonatal,1, (121200) | chemical synaptic transmission, nervous system development, regulation of ion transmembrane transport, potassium ion transmembrane transport. |
|  | Developmental and epileptic encephalopathy 7, (613720) |  |
| KMT2D | Kabuki syndrome 1, (147920) | oocyte growth, chromatin organization, regulation of transcription, DNA-templated, positive regulation of cell proliferation, heterochromatin assembly, methylation, positive regulation of intracellular estrogen receptor signaling pathway, response to estrogen, histone H3-K4 dimethylation, positive regulation of transcription from RNA polymerase II promoter, oogenesis, histone H3-K4 methylation, histone H3-K4 trimethylation, histone H3-K4 monomethylation, beta-catenin-TCF complex assembly. |
| MECP2 | Intellectual developmental disorder, X-linked, syndromic 13, (300055) | negative regulation of transcription from RNA polymerase II promoter, behavioral fear response, response to hypoxia, startle response, neurological system process involved in regulation of systemic arterial blood pressure, regulation of respiratory gaseous exchange by neurological system process, inositol metabolic process, regulation of gene expression by genetic imprinting, glutamine metabolic process, cellular biogenic amine metabolic process, mitotic spindle organization, Notch signaling pathway, synapse assembly, respiratory gaseous exchange, long-term memory, protein localization, glucocorticoid metabolic process, positive regulation of cell proliferation, adult locomotory behavior, visual learning, post-embryonic development, gene expression, negative regulation of gene expression, positive regulation of G2/M transition of mitotic cell cycle, glial cell proliferation, dendrite development, negative regulation of angiogenesis, histone methylation, histone acetylation, proprioception, sensory perception of pain, cerebellum development, ventricular system development, heterochromatin assembly, cardiolipin metabolic process, social behavior, neuron maturation, negative regulation of neuron apoptotic process, negative regulation of blood vessel endothelial cell migration, negative regulation of transcription, DNA-templated, positive regulation of transcription from RNA polymerase II promoter, phosphatidylcholine metabolic process, catecholamine secretion, negative regulation of smooth muscle cell differentiation, response to other organism, excitatory postsynaptic potential, positive regulation of glial cell proliferation, long-term synaptic potentiation, positive regulation of microtubule nucleation, positive regulation of histone H3-K9 trimethylation, positive regulation of DNA methylation, negative regulation of transcription from RNA polymerase II promoter involved in smooth muscle cell differentiation. |
|  | Intellectual developmental disorder, X-linked syndromic, Lubs type, (300260) |  |
|  | Autism susceptibility, X-linked 3, (300496) |  |
|  | Encephalopathy, neonatal severe, (300673) |  |
|  | Rett syndrome, (312750);  Rett syndrome, atypical, (312750);  Rett syndrome, preserved speech variant, (312750) |  |
| UBE3A | Angelman syndrome, (105830) | protein polyubiquitination, ovarian follicle development, proteolysis, ubiquitin-dependent protein catabolic process, brain development, positive regulation of phosphatidylinositol 3-kinase signaling, androgen receptor signaling pathway, positive regulation of protein ubiquitination, response to progesterone, sperm entry, locomotory exploration behavior, response to cocaine, response to hydrogen peroxide, regulation of circadian rhythm, positive regulation of transcription from RNA polymerase II promoter, rhythmic process, modulation of synaptic transmission, progesterone receptor signaling pathway, protein autoubiquitination, prostate gland growth, negative regulation of dendritic spine morphogenesis, motor learning, protein K48-linked ubiquitination, positive regulation of Golgi lumen acidification, cellular response to brain-derived neurotrophic factor stimulus, regulation of protein ubiquitination involved in ubiquitin-dependent protein catabolic process. |
| ARID1B | Coffin-Siris syndrome 1, (135900) | response to ischemia, chromatin remodeling, regulation of transcription from RNA polymerase II promoter, nervous system development, regulation of mitotic metaphase/anaphase transition, positive regulation of T cell differentiation, positive regulation of cell differentiation, positive regulation of myoblast differentiation, positive regulation of gene expression, epigenetic, positive regulation of transcription, DNA-templated, regulation of G0 to G1 transition, cellular response to angiotensin, regulation of G1/S transition of mitotic cell cycle, positive regulation of double-strand break repair, regulation of nucleotide-excision repair. |
| BCL11B | Immunodeficiency 49, severe combined, (617237) | keratinocyte development, epithelial cell morphogenesis, regulation of transcription from RNA polymerase II promoter, transcription from RNA polymerase II promoter, negative regulation of cell proliferation, regulation of keratinocyte proliferation, regulation of lipid metabolic process, striatal medium spiny neuron differentiation, commitment of neuronal cell to specific neuron type in forebrain, post-embryonic camera-type eye development, T cell differentiation in thymus, T cell receptor V(D)J recombination, hematopoietic stem cell migration, odontogenesis of dentin-containing tooth, negative regulation of apoptotic process, positive T cell selection, regulation of neuron differentiation, positive regulation of transcription from RNA polymerase II promoter, alpha-beta T cell differentiation, thymus development, thymocyte apoptotic process, negative regulation of thymocyte apoptotic process, olfactory bulb axon guidance, lymphoid lineage cell migration into thymus. |
|  | Intellectual developmental disorder with dysmorphic facies, speech delay, and T-cell abnormalities, (618092) |  |
| CASK | FG syndrome 4, (300422);  Intellectual developmental disorder, With or without nystagmus, (300422) | negative regulation of cell-matrix adhesion, protein phosphorylation, cell adhesion, negative regulation of keratinocyte proliferation, positive regulation of transcription from RNA polymerase II promoter, GMP metabolic process, GDP metabolic process, regulation of neurotransmitter secretion, localization, establishment of localization in cell, negative regulation of wound healing, calcium ion import, positive regulation of calcium ion import, negative regulation of cellular response to growth factor stimulus, regulation of synaptic vesicle exocytosis. |
|  | Intellectual developmental disorder and microcephaly with pontine and cerebellar hypoplasia, (300749) |  |
| CHD7 | CHARGE syndrome, (214800) | skeletal system development, in utero embryonic development, blood vessel remodeling, heart morphogenesis, ventricular trabecula myocardium morphogenesis, right ventricular compact myocardium morphogenesis, chromatin organization, chromatin remodeling, regulation of transcription, DNA-templated, rRNA processing, transcription from RNA polymerase II promoter, central nervous system development, adult heart development, sensory perception of sound, adult walking behavior, blood circulation, response to bacterium, regulation of gene expression, regulation of release of sequestered calcium ion into cytosol by sarcoplasmic reticulum, cranial nerve development, olfactory nerve development, olfactory bulb development, T cell differentiation, female genitalia development, embryonic hindlimb morphogenesis, aorta morphogenesis, atrioventricular canal development, positive regulation of multicellular organism growth, olfactory behavior, inner ear morphogenesis, chordate embryonic development, nose development, positive regulation of transcription from RNA polymerase II promoter, semicircular canal morphogenesis, genitalia development, regulation of neurogenesis, cognition, retina development in camera-type eye, regulation of growth hormone secretion, limb development, face development, innervation, cardiac septum morphogenesis, epithelium development. |
|  | Hypogonadotropic hypogonadism 5 with or without anosmia, (612370) |  |
| CNTN5 |  | axon guidance, brain development, sensory perception of sound, cell-cell adhesion, presynapse assembly. |
| CTCF | Intellectual developmental disorder, Autosomal dominant 21, (615502) | negative regulation of transcription from RNA polymerase II promoter, DNA methylation, regulation of gene expression by genetic imprinting, regulation of transcription from RNA polymerase II promoter, chromosome segregation, negative regulation of cell proliferation, maintenance of DNA methylation, positive regulation of gene expression, negative regulation of gene expression, nucleosome positioning, regulation of histone methylation, regulation of histone acetylation, regulation of gene expression, epigenetic, regulation of molecular function, epigenetic, hypermethylation of CpG island, negative regulation of transcription, DNA-templated, positive regulation of transcription, DNA-templated, regulation of centromeric sister chromatid cohesion, protein localization to chromosome, centromeric region, genetic imprinting. |
| DALRD3 | Developmental and epileptic encephalopathy 86,(618910) | arginyl-tRNA aminoacylation. |
| DEAF1 | Vulto-van Silfout-de Vries syndrome, (615828) | negative regulation of transcription from RNA polymerase II promoter, neural tube closure, regulation of transcription from RNA polymerase II promoter, transcription from RNA polymerase II promoter, germ cell development, anatomical structure morphogenesis, regulation of mammary gland epithelial cell proliferation, negative regulation of transcription, DNA-templated, positive regulation of transcription, DNA-templated, embryonic skeletal system development. |
|  | Neurodevelopmental disorder with hypotonia, impaired expressive language, and with or without seizures, (617171) |  |
| DNM1 | Developmental and epileptic encephalopathy 31,(616346) | endocytosis, receptor-mediated endocytosis, endosome organization, synaptic vesicle budding from presynaptic endocytic zone membrane, receptor internalization, synaptic vesicle endocytosis, modulation of synaptic transmission, toxin transport. |
| DYNC1H1 | Spinal muscular atrophy, lower extremity-predominant 1,AD,(158600) | mitotic cell cycle, microtubule-based movement, mitotic spindle organization, nuclear migration, retrograde axonal transport, cytoplasmic microtubule organization, positive regulation of intracellular transport, cytoplasmic mRNA processing body assembly, stress granule assembly, establishment of spindle localization, cell division, regulation of mitotic spindle organization, minus-end-directed vesicle transport along microtubule, regulation of metaphase plate congression, positive regulation of spindle assembly. |
|  | Charcot-Marie-Tooth disease, axonal, type 20, (614228) |  |
|  | Intellectual developmental disorder, autosomal dominant 13, (614563) |  |
| GABRA1 | Epilepsy, childhood absence, Susceptibility to, 4, (611136); Epilepsy, juvenile myoclonic, Susceptibility to, 5, (611136) | chloride transport, signal transduction, gamma-aminobutyric acid signaling pathway, chemical synaptic transmission, ion transmembrane transport, regulation of membrane potential, neurological system process, synaptic transmission, GABAergic, regulation of postsynaptic membrane potential, chloride transmembrane transport, inhibitory synapse assembly. |
|  | Developmental and epileptic encephalopathy 19, (615744) |  |
| GNAO1 | Developmental and epileptic encephalopathy 17, (615473) | protein folding, muscle contraction, G-protein coupled receptor signaling pathway, adenylate cyclase-modulating G-protein coupled receptor signaling pathway, dopamine receptor signaling pathway. |
|  | Neurodevelopmental disorder with involuntary movements, (617493) |  |
| GRIN1 | Neurodevelopmental disorder with or without hyperkinetic movements and seizures, autosomal dominant, (614254) | cation transport, chemical synaptic transmission, brain development, visual learning, positive regulation of calcium ion transport into cytosol, propylene metabolic process, calcium-mediated signaling, ionotropic glutamate receptor signaling pathway, response to chemical, regulation of membrane potential, response to ethanol, positive regulation of transcription from RNA polymerase II promoter, regulation of synaptic plasticity, regulation of neuronal synaptic plasticity, protein heterotetramerization, positive regulation of synaptic transmission, glutamatergic, calcium ion homeostasis, excitatory postsynaptic potential, calcium ion transmembrane import into cytosol, cation transmembrane transport, excitatory chemical synaptic transmission, positive regulation of reactive oxygen species biosynthetic process, regulation of cation transmembrane transport, response to glycine, positive regulation of excitatory postsynaptic potential, positive regulation of cysteine-type endopeptidase activity. |
|  | Neurodevelopmental disorder with or without hyperkinetic movements and seizures, autosomal recessive, (617820) |  |
|  | Developmental and epileptic encephalopathy 101, (619814) |  |
| KIDINS220 | Spastic paraplegia, intellectual disability, nystagmus, and obesity, (617296) | in utero embryonic development, positive regulation of neuron projection development, nerve growth factor signaling pathway, dendrite morphogenesis, cellular response to nerve growth factor stimulus. |
|  | Ventriculomegaly and arthrogryposis, (619501) |  |
| KLHL17 |  | brain development, protein ubiquitination, actin cytoskeleton organization. |
| L1CAM | CRASH syndrome, (303350);  MASA syndrome, (303350) | chemotaxis, cell adhesion, homophilic cell adhesion via plasma membrane adhesion molecules, cell-matrix adhesion, nervous system development, axon guidance, cell migration, neuron projection development, positive regulation of axon extension, synapse organization, axon development. |
|  | Corpus callosum, partial agenesis of, (304100) |  |
|  | Hydrocephalus due to aqueductal stenosis, (307000);  Hydrocephalus with Hirschsprung disease, (307000);  Hydrocephalus with congenital idiopathic intestinal pseudoobstruction, (307000) |  |
| MMACHC | Methylmalonic aciduria and homocystinuria, cblC type, (277400) | glutathione metabolic process, cell surface receptor signaling pathway, cobalamin metabolic process, regulation of signaling, demethylation. |
| MUC4 |  | cell-matrix adhesion, regulation of receptor activity, maintenance of gastrointestinal epithelium. |
| MUC6 |  | maintenance of gastrointestinal epithelium. |
| RIN2 | Macrocephaly, alopecia, cutis laxa, and scoliosis, (613075) | endocytosis, signal transduction, small GTPase mediated signal transduction, positive regulation of endothelial cell migration, vesicle-mediated transport, positive regulation of endothelial cell-matrix adhesion via fibronectin, positive regulation of vasculogenesis. |
| SATB2 | Glass syndrome, (612313) | negative regulation of transcription from RNA polymerase II promoter, neuron migration, osteoblast development, chromatin remodeling, regulation of transcription from RNA polymerase II promoter, embryonic pattern specification, commitment of neuronal cell to specific neuron type in forebrain, positive regulation of transcription from RNA polymerase II promoter, embryonic skeletal system morphogenesis, cartilage development, palate development, cellular response to organic substance |
| SATL1 |  | spermidine acetylation. |
| SCN8A | Myoclonus, familial, 2, (618364)  Cognitive impairment with or without cerebellar ataxia, ( 614306)  Developmental and epileptic encephalopathy 13, (614558)  Seizures, benign familial infantile, 5, (617080) |  |
| SLC6A8 | Cerebral creatine deficiency syndrome 1, (300352) | creatine metabolic process, neurotransmitter transport, muscle contraction, creatine transport, sodium ion transmembrane transport, organic substance transport, nitrogen compound transport. |
| SMC1A | Cornelia de Lange syndrome 2, (300590)  Developmental and epileptic encephalopathy 85, with or without midline brain defects, (301044) | mitotic sister chromatid segregation, DNA repair, sister chromatid cohesion, mitotic sister chromatid cohesion, response to radiation, stem cell population maintenance, establishment of mitotic sister chromatid cohesion, establishment of meiotic sister chromatid cohesion, somatic stem cell population maintenance, cell division, meiotic cell cycle, response to DNA damage checkpoint signaling, mitotic spindle assembly. |
| STXBP1 | Developmental and epileptic encephalopathy 4, (612164) | platelet degranulation, developmental process involved in reproduction, intracellular protein transport, vesicle docking involved in exocytosis, neurotransmitter secretion, neuromuscular synaptic transmission, axon target recognition, regulation of synaptic vesicle priming, protein transport, synaptic vesicle priming, synaptic vesicle maturation, vesicle-mediated transport, negative regulation of protein complex assembly, regulation of synaptic vesicle fusion to presynaptic membrane, negative regulation of synaptic transmission, GABAergic, response to estradiol, SNARE complex assembly, regulation of SNARE complex assembly, positive regulation of mast cell degranulation, negative regulation of neuron apoptotic process, positive regulation of calcium ion-dependent exocytosis, protein stabilization, neuron apoptotic process, long term synaptic depression, platelet aggregation, cellular response to interferon-gamma, protein localization to plasma membrane, presynaptic dense core vesicle exocytosis, positive regulation of glutamate secretion, neurotransmission, regulation of acrosomal vesicle exocytosis. |
| PAH | Hyperphenylalaninemia, non-PKU mild, (261600)  Phenylketonuria, (261600) | L-phenylalanine catabolic process, tyrosine biosynthetic process, cellular amino acid biosynthetic process, aromatic amino acid family metabolic process, tyrosine biosynthetic process, by oxidation of phenylalanine, neurotransmitter biosynthetic process, catecholamine biosynthetic process. |
