# Supplementary material for: Genetic and phenotypic analysis of 225 Chinese children with developmental delay and/or intellectual disability using whole-exome sequencing
Source: BMC Genomics. 2024 Apr 22;25:391. doi: 10.1186/s12864-024-10279-1 (PMC11034079; doi:10.1186/s12864-024-10279-1)
Supplement: Supplementary file 6 — Supplementary Material 6 [file 12864_2024_10279_MOESM6_ESM.docx]

**Supplementary Table 7** Different diagnostic yields of WES in the three WES laboratories

| WES laboratories | Individuals  (n(%)) | P/LP diagnosed individuals (n(%)) | P value |
| --- | --- | --- | --- |
| AGMT ^a^ | 128(56.89) | 55(42.97) |  |
| KMD ^b^ | 69(30.67) | 28(40.58) |  |
| RGI ^c^ | 28(12.44) | 13(46.43) |  |
| **Total** | 225(100) | 96(42.67) | 0.865 |

^a^ Angen Gene Medicine Tech (Beijing, China)

^b^ Kaiumph Medical Diagnostic Lo. Ltd (Beijing, China)

^c^ Running Gene Inc. (Beijing, China)
