# Supplementary material for: Genetic and phenotypic analysis of 225 Chinese children with developmental delay and/or intellectual disability using whole-exome sequencing
Source: BMC Genomics. 2024 Apr 22;25:391. doi: 10.1186/s12864-024-10279-1 (PMC11034079; doi:10.1186/s12864-024-10279-1)
Supplement: Supplementary file 7 — Supplementary Material 7 [file 12864_2024_10279_MOESM7_ESM.docx]

**Supplementary Table 4** Impact of WES on medical management

| Patient number | Gene | Ending a diagnostic "odyssey" | Reproductive decision changes | Medical care changes | | | | Outcome：normal development/  significantly improved |
| --- | --- | --- | --- | --- | --- | --- | --- | --- |
|  |  |  |  | initiation of disease monitoring/systemic investigation | discontinuation medication | Addition of medication | Physiotherapy/psychosocial supports |  |
| Patient 004 | KRIT1 |  |  |  |  |  |  |  |
| Patient 007 | KLHL17 |  |  |  |  |  |  |  |
| Patient 008 | BCL11B |  |  |  |  | + |  |  |
| Patient 010 | ASXL3 |  |  |  |  |  |  |  |
| Patient 012 | BRPF1 |  |  |  |  | + | + | + |
| Patient 013 | CNTN5 |  |  |  |  |  |  |  |
| Patient 014 | ABCB5 |  |  |  |  | + |  |  |
| Patient 015 | SLC6A8 |  |  |  |  | + | + | + |
| Patient 017 | CHD2 |  | + |  |  | + | + | + |
| Patient 021 | GLI2 |  |  | + |  | + | + | + |
| Patient 024 | GRIN2A |  |  |  |  |  |  |  |
| Patient 028 | VPS13B | + | + |  |  |  |  |  |
| Patient 029 | VPS13B | + | + |  |  |  |  |  |
| Patient 031 | SMARCA4 |  |  |  |  |  |  |  |
| Patient 032 | AHDC1 |  |  |  |  |  |  |  |
| Patient 033 | ASXL3 |  |  |  |  |  |  |  |
| Patient 036 | UBE3A |  | + |  |  | + |  |  |
| Patient 037 | UBE3A | + |  |  |  |  |  |  |
| Patient 038 | MECP2 |  | + |  |  |  |  |  |
| Patient 039 | MECP2 |  | + |  |  |  |  |  |
| Patient 040 | GRIN1 |  |  |  |  |  |  |  |
| Patient 042 | ZNF597 |  |  |  |  |  |  |  |
| Patient 043 | COL4A5 |  | + |  |  | + |  |  |
| Patient 047 | CASK | + | + |  |  |  |  |  |
| Patient 048 | CASK | + | + |  |  |  |  |  |
| Patient 062 | FOXP1 |  |  |  |  |  |  |  |
| Patient 063 | P4HTM |  |  |  |  |  |  |  |
| Patient 067 | CDKL5 |  | + |  |  | + | + |  |
| Patient 068 | EIF4G1、HSPB1 |  |  |  |  |  |  |  |
| Patient 069 | HDAC4 |  |  |  |  |  |  |  |
| Patient 071 | NKX2-1 |  |  | + |  |  |  |  |
| Patient 076 | MMACHC |  | + |  |  | + |  |  |
| Patient 079 | KCNQ2 |  |  |  | + | + |  |  |
| Patient 080 | MECP2 |  |  |  |  |  |  |  |
| Patient 081 | SATL1 |  |  |  |  |  |  |  |
| Patient 082 | DALRD3 |  |  |  |  |  |  |  |
| Patient 089 | MUC4 |  |  |  |  |  |  |  |
| Patient 091 | DEAF1 |  |  |  |  |  |  |  |
| Patient 092 | KIDINS220 |  |  |  |  |  |  |  |
| Patient 097 | KIDINS220 |  |  |  |  |  |  |  |
| Patient 100 | KMT2D |  |  |  |  |  |  |  |
| Patient 104 | ATP6AP1 |  |  |  |  |  |  |  |
| Patient 106 | SMARCA2 |  |  |  |  |  |  |  |
| Patient 107 | GLI3 |  |  |  | + |  | + |  |
| Patient 110 | GABRA1 |  |  |  |  | + |  |  |
| Patient 114 | DYNC1H1 |  |  |  |  |  |  |  |
| Patient 115 | IQSEC2 |  | + |  |  | + |  |  |
| Patient 116 | IQSEC2 |  | + |  |  | + |  |  |
| Patient 117 | CTCF |  |  |  |  |  |  |  |
| Patient 118 | NESTIN |  |  |  |  |  |  |  |
| Patient 119 | STXBP1 |  |  |  |  | + | + | + |
| Patient 124 | RIN2 |  |  |  |  |  |  |  |
| Patient 125 | ARID1B |  |  |  |  |  |  |  |
| Patient 129 | PTEN |  |  |  |  |  |  |  |
| Patient 131 | ARID1B |  |  |  |  |  |  |  |
| Patient 132 | HNRNPH2 |  |  |  |  |  |  |  |
| Patient 133 | TH |  | + |  | + | + |  |  |
| Patient 134 | UBE3B |  | + |  |  | + |  |  |
| Patient 137 | SCN9A |  | + |  |  | + |  |  |
| Patient 141 | EEF1A2 |  |  |  |  |  |  |  |
| Patient 142 | PAH、SATB2 |  | + |  |  | + |  |  |
| Patient 147 | ZEB2 |  |  |  |  |  |  |  |
| Patient 148 | CHD7 |  |  | + |  |  |  |  |
| Patient 150 | B3GALNT2 |  | + |  |  | + |  | + |
| Patient 151 | GABRB3 |  |  |  | + | + |  |  |
| Patient 152 | EFTUD2 |  |  |  |  |  |  |  |
| Patient 153 | DNM1 |  |  |  |  | + | + | + |
| Patient 154 | SMC1A |  |  |  |  | + |  | + |
| Patient 156 | MAT1A |  |  |  |  | + | + | + |
| Patient 158 | MUC6 |  |  |  |  |  |  |  |
| Patient 159 | NOTCH1 |  |  |  |  |  |  |  |
| Patient 162 | YAP1 |  |  |  |  |  |  |  |
| Patient 169 | CUX2 |  | + |  |  |  |  |  |
| Patient 171 | FGD1 |  | + |  |  |  |  |  |
| Patient 175 | BRPF1 |  |  |  |  | + |  | + |
| Patient 176 | MEF2C |  |  |  |  |  |  |  |
| Patient 179 | HNRNPH2 |  |  |  |  |  |  |  |
| Patient 180 | TBX6 |  |  |  | + |  |  |  |
| Patient 181 | UBE3A |  |  |  |  |  |  |  |
| Patient 182 | UBE3A |  |  |  |  |  |  |  |
| Patient 187 | KRT9、SETD2、PRDM16 |  |  |  |  |  |  |  |
| Patient 195 | KCNQ2 |  |  |  | + | + | + | + |
| Patient 197 | FOXP1 |  |  |  |  |  |  |  |
| Patient 198 | L1CAM |  |  |  |  |  |  |  |
| Patient 200 | GNAO1 |  |  |  |  | + |  |  |
| Patient 201 | ACTA2 | + | + |  |  |  |  |  |
| Patient 207 | KMT2D |  |  |  |  |  |  |  |
| Patient 208 | ACTG1 |  |  | + |  |  |  |  |
| Patient 210 | MMACHC |  | + |  |  | + | + | + |
| Patient 214 | ASXL3 |  |  |  |  |  |  |  |
| Patient 220 | SYNGAP1 |  |  |  |  | + | + |  |
| Patient 221 | SON |  |  |  |  |  |  |  |
| Patient 222 | GRIN2B |  |  |  |  |  |  |  |
| Patient 223 | ASXL3 |  |  |  |  |  |  |  |
| Patient 224 | ATP1A3 |  |  | + |  | + |  |  |
| Patient 225 | CLCN4 |  |  |  |  |  |  |  |
| Total |  | 6 | 22 | 35 | | | | 12 |
